# Surgical Interventions for Rotator Cuff Tears: A Comprehensive Literature Review

**DOI:** 10.7759/cureus.75606

**Published:** 2024-12-12

**Authors:** Abdirizak Aden, Sultan Alsayeh, Lena Aburas, Qazi Masood

**Affiliations:** 1 Trauma and Orthopedics, Medway Maritime Hospital, Gillingham, GBR

**Keywords:** arthroscopic rotator cuff repair, open rotator cuff repair, patient outcomes, rotator cuff tear, surgical interventions

## Abstract

Rotator cuff tears in the shoulder joint are common musculoskeletal injuries that may present with or without symptoms. Rotator cuff tears are a common musculoskeletal condition that become increasingly prevalent with age.

This mines various surgical interventions for rotator cuff tears, focusing on patient selection criteria and treatment outcomes across different subgroups. A comprehensive search was conducted to identify studies on rotator cuff tear classifications and surgical techniques. The review analyzes patient characteristics influencing treatment choices and outcomes.

Rotator cuff tears are classified by size and extent. Surgical interventions range from arthroscopic repairs to reverse shoulder arthroplasty (RSA), each with specific indications. Patient factors such as age, tear size, activity level, and overall health influence the selection of surgical technique. Minimally invasive procedures generally offer reduced times and complications, while open surgeries may be necessary for complex tears.

The choice of surgical treatment for rotator cuff tears is determined by multiple factors, including tear characteristics and patient demographics. Tailoring interventions to individual patient needs can optimize outcomes and restore shoulder function effectively.

## Introduction and background

Introduction

Tears of the rotator cuff in the shoulder joint are a prevalent musculoskeletal injury that may or may not present symptoms. The prevalence of rotator cuff tears in the general population exceeds 20% and is recognized to increase with age [1].

A systematic analysis found that the prevalence of full-thickness rotator cuff tears in symptomatic individuals aged 44-50 years was 35% when evaluated by ultrasonography and 41% when analyzed using magnetic resonance imaging (MRI) [2].

Treatment options include both surgical and non-surgical (conservative) methods. The primary objective of both approaches is to alleviate pain and restore shoulder functionality. The surgical management of rotator cuff tears involves various techniques tailored to the specific characteristics of the injury, patient factors, and surgeon preferences [3].

Classification of rotator cuff tears

Rotator cuff tears are categorized according to their size (partial or complete), using various classification methods. Partial tears are primarily classified based on the extent of tearing at the footprint, as per the Ellman classification observed on MRI. Full-thickness tears are often categorized using the Codman classification, which considers the size of the tear in the anterior-posterior dimension (Table 1) [1].

**Table 1:** Classification of rotator cuff tears

Partial-thickness tears, Ellman classification [4]	
Grade 1	<3 mm (<25% thickness)
Grade 2	3-6 mm (25%-50% thickness)
Grade 3	>6 mm (>50% thickness)
Location: A, articular sided; B, bursal sided; and C, intratendinous	
Full-thickness tears, Codman classification (tear size in the anterior to posterior direction) [5]	
Small	0-1 cm
Medium	1-3 cm
Large	3-5 cm
Massive	>5 cm

## Review

Surgical interventions in rotator cuff tears

The surgical management of rotator cuff tears involves various techniques tailored to the specific characteristics of the injury, patient factors, and surgeon preferences. In most circumstances, patients should typically undergo at least 3-6 months of non-surgical treatment before considering surgery, unless there is an acute, traumatic tear [3].

Arthroscopic Rotator Cuff Repair

Technique: This minimally invasive technique uses small incisions and an arthroscope to visualize the shoulder joint. The surgeon reconstructs the ruptured tendon using anchors and sutures, reattaching it to the humeral head. The initial procedure for rotator cuff tendon-bone reattachment was single-row repair; however, due to its limited capacity to restore the anatomical footprint, double-row repair configurations were subsequently devised [6]. Initial double-row arrangements featured medial mattress sutures that were not connected to lateral row sutures. Recently, bridge-type constructions connect both medial and lateral row anchors, enhancing tendon-bone compression, construct stiffness, failure load, and gap resistance [7].

Patient selection: Patients with full-thickness rotator cuff tears, especially those that are medium to large in size, are typically considered for arthroscopic repair. Partial-thickness tears may also be addressed, particularly if they cause significant symptoms. While younger, active patients often have better outcomes, older patients can also be considered if they have reasonable expectations and no significant comorbidities [7].

Benefits: Arthroscopic repair minimizes incisions, reduces pain, shortens recovery periods, and decreases the risk of complications relative to open surgery. Compared to single-row repairs, double-row repair generally yields a superior healing rate. A double-row repair is preferred unless it leads to a high-tension repair. Over-tensioned double-row repairs can occur with retracted, stationary tears and may result in rotator cuff failure at the musculotendinous junction, medial to the suture fixation. A meticulously conducted single-row repair may be favored to prevent this. Moreover, the minimally invasive nature reduces the risk of complications such as infection, blood loss, and stiffness, which are more common with open repairs [8].

Disadvantages: Some surgeons may have more experience with open techniques, and arthroscopy requires specialized training and skill. Less experienced surgeons may face challenges that could affect outcomes. The smaller surgical field can make it more difficult to visualize and access certain areas of the rotator cuff or address more complex injuries. While generally successful, there is still a risk of re-tearing the rotator cuff, especially in elderly patients or those with large tears. Although less frequent than in open surgery, some patients may still experience significant pain or stiffness in the shoulder after the procedure, requiring rehabilitation and physical therapy [6].

Open Rotator Cuff Repair

Technique: A larger incision is made to allow direct access to the torn rotator cuff. This method may be preferred for larger or more complex tears or cases where significant scarring exists. The torn tendon is examined for the extent of the tear. If there is retraction or significant degeneration, additional procedures may be needed, such as the debridement of damaged tissue. The torn ends of the rotator cuff are reattached to the greater tuberosity of the humerus using sutures and anchors to secure the tendon in place [9].

Patient selection: Open repair is often considered for patients with larger or more complex full-thickness rotator cuff tears, particularly those that are irreparable by arthroscopic methods. Larger and more retracted tears, or those evaluated as having significant muscle belly atrophy, may necessitate open repair for better exposure and repair success. Moreover, in older patients who may have degenerative changes and are less likely to benefit from minimally invasive techniques, open repair may be preferred for more direct access and repair [9].

Benefits: Open repair provides better visualization and access to the tissues involved, which may facilitate repair in complicated cases. The surgeon can perform more extensive debridement and address associated problems (such as subacromial impingement) more effectively. Additionally, for larger tears, open repair may have lower re-tear rates compared to arthroscopic repairs [10].

Disadvantages: The larger incision and dissection result in more trauma to the surrounding tissues, leading to increased pain and a longer recovery time compared to arthroscopic repair. The surgical trauma associated with a larger incision can result in higher levels of postoperative pain, requiring more intensive pain management. Furthermore, the larger incision can cause noticeable scarring and a higher risk of postoperative complications, such as infection, blood loss, stiffness, and nerve or blood vessel injury [1].

Mini-Open Rotator Cuff Repair

Technique: This hybrid technique combines elements of both arthroscopic and open repairs. It involves a small incision (larger than arthroscopic but smaller than traditional open techniques) to repair the tendon after arthroscopic visualization. It is ideal for tears that are difficult to repair using only arthroscopic methods but do not require full open repair [9].

Patient selection: Mini-open repair is often indicated for patients with medium to large full-thickness rotator cuff tears that may not be amenable to complete arthroscopic repair. In some cases, partial-thickness tears that cause significant symptoms or functional limitations may also be treated with mini-open repair.

Benefits: This technique balances the advantages of both arthroscopic and open approaches, allowing for adequate visualization while minimizing tissue trauma. It is less invasive than traditional open repair, resulting in reduced complications, faster recovery, and easier rehabilitation [11].

Disadvantages: While less invasive than a traditional open repair, a mini-open surgery still provides less exposure than a full open approach, which may make it challenging for certain complex repairs. Additionally, surgeons must be skilled in both arthroscopic and open techniques, and not all surgeons may offer this approach. Therefore, outcomes can vary based on factors such as tear size, patient age, and overall health and may not be universally superior to either traditional open or purely arthroscopic repairs [12].

Transosseous Equivalent Repair

Technique: This method involves creating suture tunnels through the bone to secure the tendon to the humerus, providing strong fixation. It enhances the ability to restore the footprint of the rotator cuff on the bone and aims to replicate the native muscle-tendon-bone interface while enhancing the repair mechanics. Instead of using traditional anchors, the transosseous technique involves drilling holes into the greater tuberosity of the humerus to create tunnels for the sutures. Heavy-weight, nonabsorbable sutures are passed through the tendon and these bone tunnels. This process creates a "suture bridge" that mimics the natural tendon-bone connection by reattaching the tendons directly to the bone. The sutures are tied outside the shoulder, securing the tendon to the bone while allowing for tension adjustments [13].

Patient selection: Patients with large or massive full-thickness rotator cuff tears are often the best candidates for transosseous repairs because this technique provides enhanced fixation and minimizes stress on the repair. While transosseous repairs can benefit a wide range of age groups, younger, more active patients may see significant advantages in terms of functional recovery. However, older patients with significant tendon degeneration or comorbidities might still be candidates [13].

Benefits: This technique promotes biological healing at the tendon-bone interface, enhancing the integration of the repaired tendon with the bone. By avoiding the use of anchors, it reduces potential complications associated with anchor placement, such as anchor migration or breakage. Additionally, the suture bridge design allows for better load distribution across the tendon, reducing the risk of re-tear. The stable fixation it provides is particularly beneficial for larger and more complex tears, promoting improved healing outcomes [14].

Disadvantages: The procedure requires a high level of surgical skill and experience. It can take longer to perform than traditional anchor-based repairs, increasing the total surgical time. Achieving perfect tendon-bone compression can also be challenging, particularly for certain tear configurations, which may impact healing outcomes [13].

Tendon Grafting

Technique: Tendon grafting repair is a surgical intervention used for managing significant rotator cuff tears, particularly when the remaining tendon is insufficient or of poor quality for direct repair. This technique involves using a graft (either an autograft from the patient or an allograft from a donor) to reconstruct the torn rotator cuff. The graft is secured to the rotator cuff remnant and/or the greater tuberosity of the humerus using sutures or anchors. The goal is to restore continuity and function to the shoulder [15].

Patient selection: Patients with large (>5 cm) or massive rotator cuff tears that cannot be adequately repaired due to significant retraction or the poor quality of the remaining tendon tissue are prime candidates for tendon grafting [15].

Benefits: Grafting is beneficial for large or irreparable tears where native tissue is insufficient, allowing for the restoration of shoulder function. The use of a graft provides additional stability to areas at risk for re-tear. Furthermore, the graft stimulates biological healing, leading to better integration with the host tissue over time [16].

Disadvantages: Tendon grafting is a complex procedure requiring significant surgical skill and experience. It often takes longer than standard rotator cuff repairs, and the rehabilitation process is more demanding and prolonged, requiring careful management to prevent graft failure. Additionally, an extra surgical site (for autograft harvesting) or the network created by the graft could lead to postoperative scar tissue, which may affect the range of motion [17].

Lateral Anchor Technique

Technique: The lateral anchor technique is a surgical approach used to repair rotator cuff tears, particularly those involving the supraspinatus tendon. This method emphasizes the placement of anchors laterally on the greater tuberosity, facilitating the restoration of tendon function and improving shoulder joint mechanics. Anchors are inserted into the lateral aspect of the greater tuberosity to enhance biomechanical stability by optimizing suture tension and load distribution [10].

Benefits: The lateral placement of anchors offers better mechanical stability, reducing the risk of re-tear through enhanced load distribution. By restoring the native anatomy and biomechanical function of the rotator cuff, patients may experience improved pain relief and shoulder function postoperatively. Additionally, this technique often minimizes trauma to surrounding tissues, promoting quicker recovery and less postoperative pain. It can be used for various sizes and types of rotator cuff tears, making it a flexible option for surgeons [14].

Patient selection: Ideal candidates typically have full-thickness rotator cuff tears (small to medium, usually ≤3 cm), particularly those involving the supraspinatus or infraspinatus tendons. The lateral anchor technique is well-suited for tears that can be adequately mobilized and repaired without extensive retraction or degradation [14].

Disadvantages: The technique can be technically challenging and requires experience to achieve optimal placement and tensioning of the anchors. The effectiveness of this technique may depend on the individual patient's anatomy, and modifications may be necessary for certain tear configurations. Potential complications associated with anchor placement, including anchor failure, migration, and soft tissue irritation, must be considered [10].

Superior Capsule Reconstruction (SCR)

Technique: Superior capsule reconstruction (SCR) is a surgical technique designed to address irreparable rotator cuff tears, particularly those involving the supraspinatus and infraspinatus tendons. This technique aims to restore shoulder stability and function by reconstructing the superior shoulder capsule using a graft [18].

Patient selection: Candidates typically present with massive, irreparable rotator cuff tears, particularly those involving the supraspinatus tendon that cannot be repaired with traditional techniques due to significant tendon retraction or degenerative changes. Most patients suitable for SCR are typically middle-aged to older adults (often 50-70 years) [18].

Benefits: SCR is designed to stabilize the glenohumeral joint, reducing the risk of superior humeral head migration and improving shoulder mechanics. The technique can help restore or enhance shoulder function, including strength and range of motion, in patients with massive, irreparable tears. Unlike some traditional repairs that may over-tension or alter the biomechanics of existing tissues, SCR focuses on augmenting the native structures of the shoulder [19].

Disadvantages: The SCR procedure requires advanced surgical skills and experience. Compared to simple rotator cuff repairs, SCR can take longer due to the additional steps involved, particularly in graft preparation and fixation. The rehabilitation period can also be extensive and complex, often requiring careful management to achieve optimal outcomes and avoid complications such as joint stiffness [20].

Reverse Shoulder Arthroplasty (RSA)

Technique: Reverse shoulder arthroplasty (RSA) is a surgical procedure designed primarily for patients with severe rotator cuff tears, especially when accompanied by shoulder arthritis or a loss of normal shoulder function. The RSA technique involves a unique design that reverses the normal anatomy of the shoulder joint, allowing for improved function and pain relief in specific patient populations. In RSA, the design of the implant places the ball (humeral component) on the shoulder blade (scapula) and the cup (glenoid component) on the humerus. This reversal is key to improving function in patients with deficient rotator cuff muscles [21].

Patient selection: Candidates typically have massive, irreparable rotator cuff tears, particularly involving the supraspinatus and infraspinatus muscles. Additionally, patients with significant glenohumeral joint arthritis due to chronic rotator cuff deficiency and those experiencing debilitating shoulder pain that limits daily activities, including overhead movements, are prime candidates. RSA is most commonly indicated for middle-aged to older patients (generally over 65 years), especially those with low functional expectations for demanding activities [21].

Benefits: RSA can restore shoulder function and improve range of motion, allowing patients to regain the ability to perform daily tasks. Because RSA alters the mechanics of the shoulder joint, it can compensate for the absence or dysfunction of the rotator cuff muscles, which is especially helpful in irreparable tears. The design reduces the risk of shoulder instability, which can be an issue in traditional shoulder arthroplasty when rotator cuff function is compromised [22].

Disadvantages: RSA is a more complex procedure than standard shoulder arthroplasty and requires specialized surgical skills and experience. While RSA can improve function, some patients may still experience a limited range of motion, particularly in overhead activities. Additionally, RSA places greater reliance on the deltoid muscle for shoulder movement, which can lead to discomfort or fatigue in that muscle postoperatively. Recovery and rehabilitation can also be extensive, often requiring a structured physical therapy program to regain strength and mobility [23].

## Conclusions

The selection of a surgical therapy strategy for rotator cuff injuries depends on multiple criteria. Each surgical procedure has specific indications, advantages, and associated risks. The choice of procedure is influenced by the dimensions and location of the tear, as well as the patient's age, activity level, and overall health. A thorough consultation with an orthopedic specialist is essential to determine the optimal strategy for addressing rotator cuff injuries.
